# Rarest of the Rare Metastatic Tubulocystic Carcinoma of Kidney

**DOI:** 10.7759/cureus.12117

**Published:** 2020-12-17

**Authors:** Hira Yousuf, Shiyam Kumar, Mansour Al-Moundhri

**Affiliations:** 1 Oncology, Pinderfields General Hospital, Wakefield, GBR; 2 Medical Oncology, Yeovil District Hospital NHS Foundation Trust, Yeovil, GBR; 3 Medical Oncology, Sultan Qaboos University Hospital, Muscat, OMN

**Keywords:** case report, tubulocystic carcinoma of the kidney, cd10 and p504s, gemcitabine with cisplatin/carboplatin, transitional cell carcinoma

## Abstract

Tubulocystic carcinoma of the kidney is a rare neoplasm with <100 case reports. Patients are usually asymptomatic and have a relatively indolent disease course occurring predominantly in males. These tumors rarely metastasize. It was previously considered to have some similarities to various other renal cancers, although this tumor has distinct macroscopic, microscopic, and immunohistochemical features. It is now a well-established entity in renal neoplastic pathology. Herein we present a case of metastatic tubulocystic carcinoma presenting with bony metastasis.

## Introduction

Tubulocystic carcinoma of the kidney is a rare neoplasm with a benign course and rarely metastasizes. It shares several similarities with other renal tumors but has a distinct macroscopic, microscopic, and immunohistochemical feature. Furthermore, it has been recognized as a distinct entity in the 2012 Vancouver classification of renal tumors [1]. It can occur both in native and transplant kidneys. However clinical presentation remains the same. The most common symptoms are abdominal pain, distension, and hematuria. Occasionally it metastasizes to lymph node, liver, bone, and pleura. The overall prognosis of the localized tumor is favorable and mainstay of treatment is nephrectomy. However, the prognosis after metastasis is dismissal owing to aggressive behavior and there is no established salvage treatment available. Treatment of the metastatic disease is based on principles of papillary renal cell carcinoma which includes chemotherapy with gemcitabine, cisplatin/carboplatin.

## Case presentation

A 43-year-old male with a history of radical nephrectomy in 2002 for (Tumor, Node, Metastasis staging) pT3a N0M0 poorly differentiated right kidney neoplasm that appeared to arise unusually from the collecting duct with sarcomatoid change. Post-surgery he was on regular follow up and remained asymptomatic and his imaging including CT scan and positron emission tomography (PET) scans did not show any evidence of recurrence. In August 2018, he presented with complaints of right shoulder and arm pain with restriction of movement for six months. Right shoulder joint pain was nonradiating, gradual onset with severity of 4/10, aggravated by movements and partially relieved by analgesia. The patient denied any other joint involvement. Rest of the systemic review was unremarkable. His general physical and systemic examination was normal. His right shoulder joint movements were restricted especially abduction and he could not abduct his arm more than 90 degrees. He also has two swellings over the scalp on the right side.

Diagnosis

**Figure 1:**
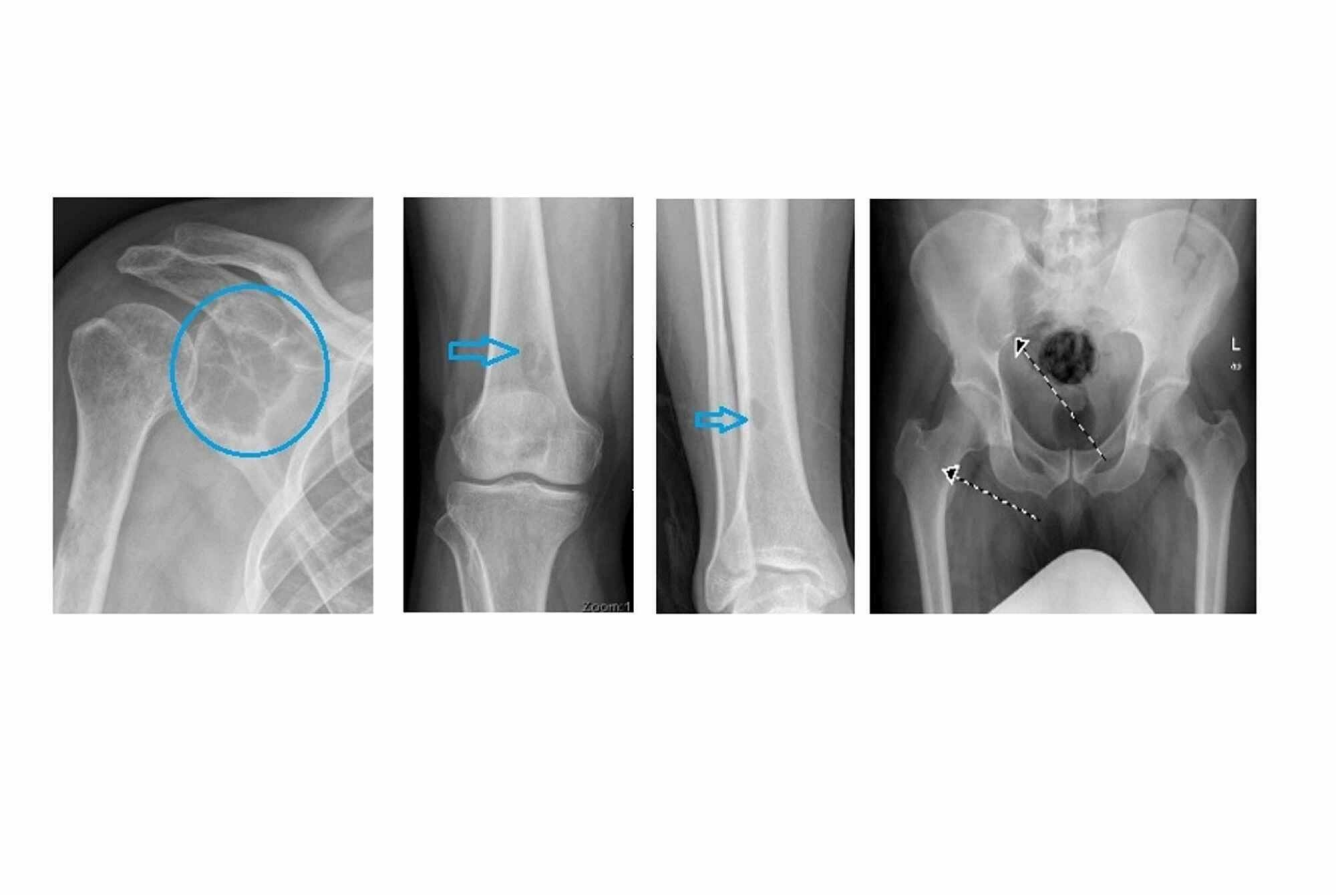
X-ray shoulder (circle) and skeletal survey (blue and black arrows) shows lytic lesions.

On the basis of his symptoms, an X-ray for shoulder joint along with skeletal survey was advised (Figure 1) which showed lytic lesions in the right humerus, right distal femur, and right iliac bone.

**Figure 2:**
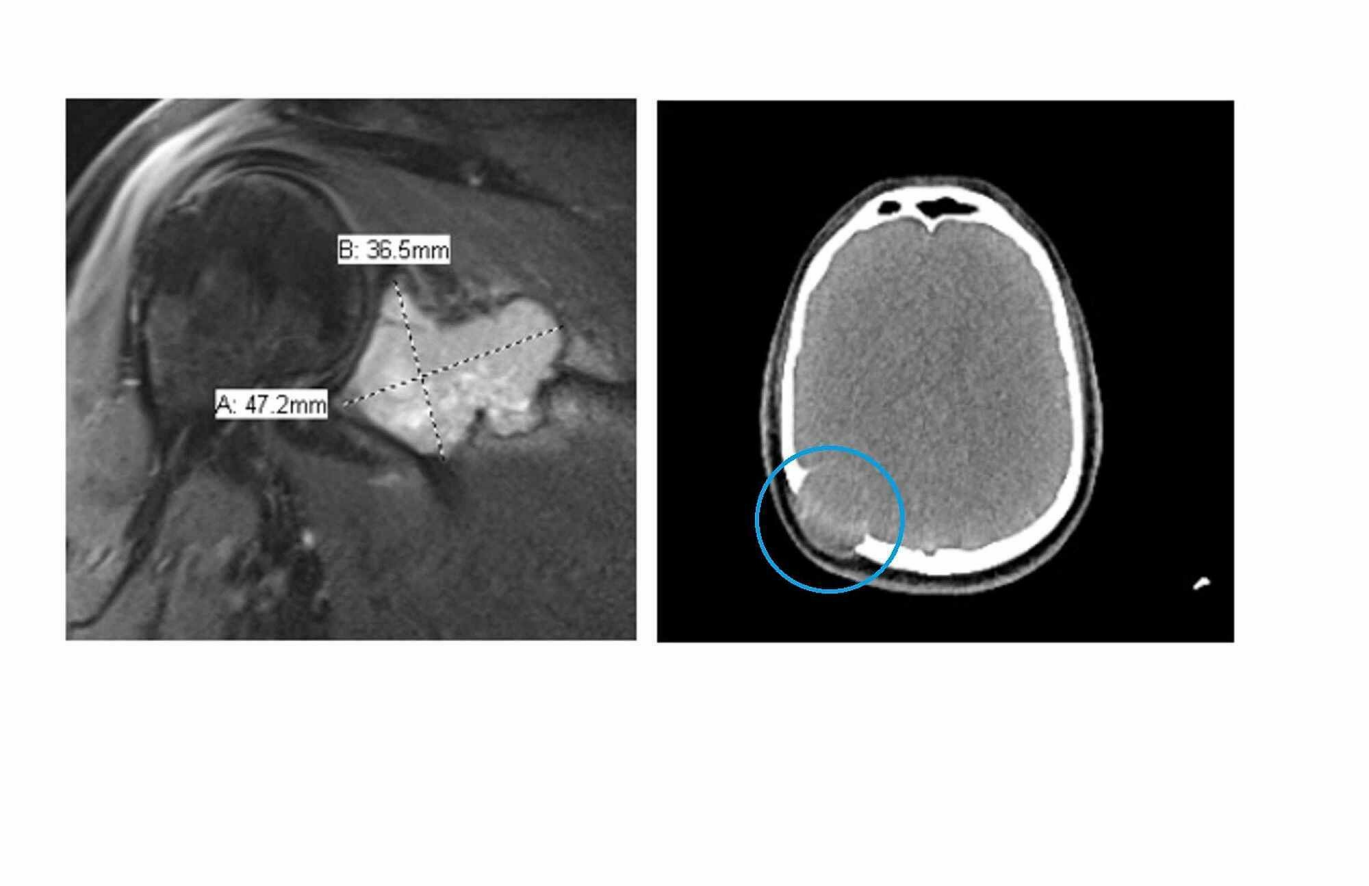
MRI shoulder shows lytic lesion. CT-Brain shows parieto-occipital expansible lesion (blue circle).

He subsequently had an MRI of the right shoulder joint (Figure 2), which showed scapular soft tissue mass, humeral lesion showing significant uptake, suggestive of metastatic disease. The patient was hospitalized to the ward for further evaluation and pain management.

**Figure 3:**
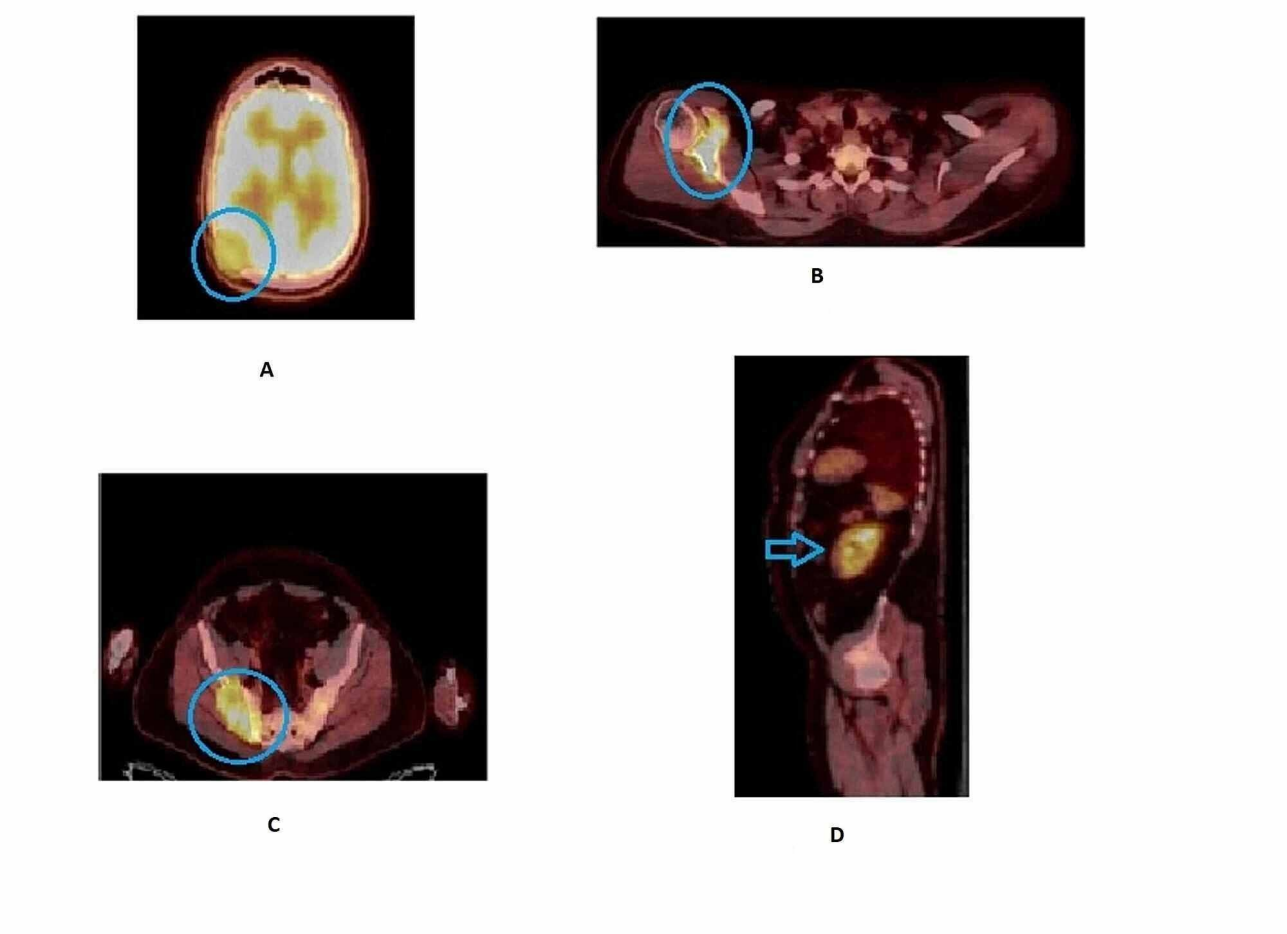
(A-D) PET-Scan shows enhanced activity indicating metastatic lesion (blue circle) occipitoparietal area (A) humerus (B) sacrum (C) and in kidney (arrowhead) (D).

**Figure 4:**
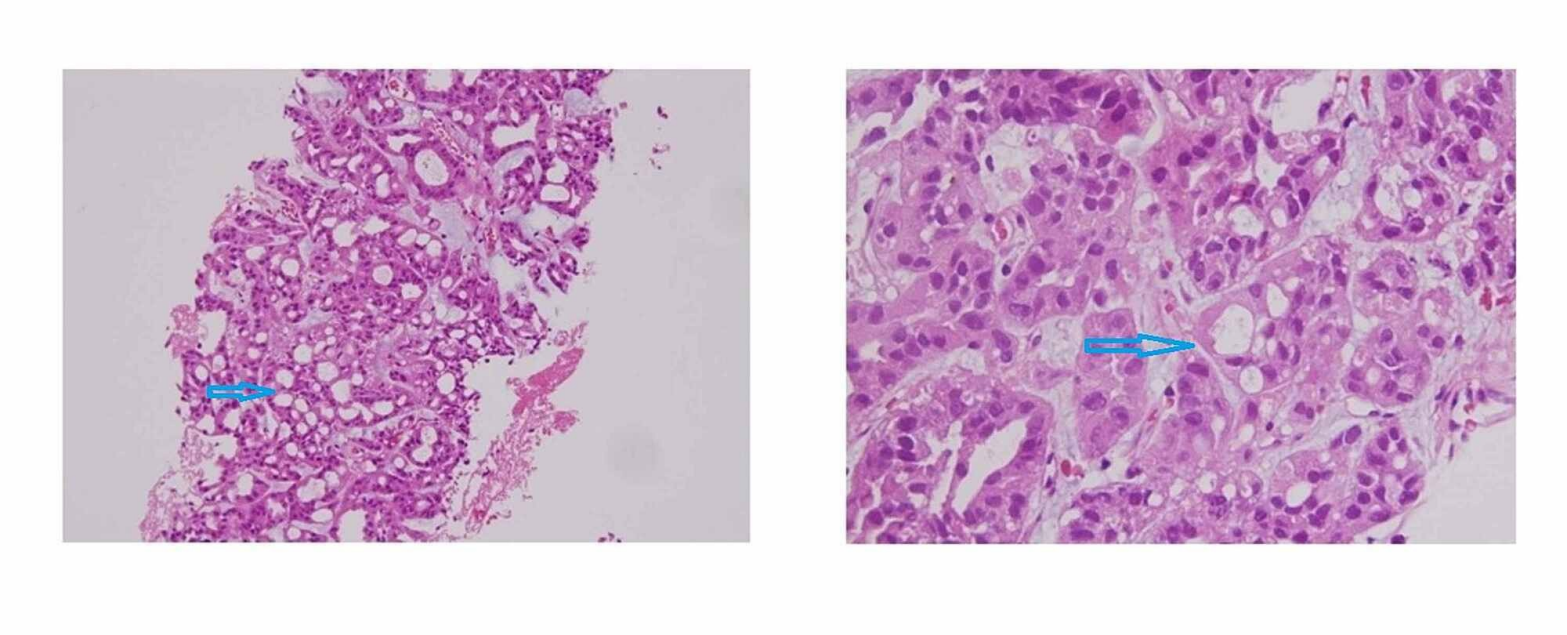
Metastatic renal tubulocystic carcinoma (arrowheads).

To assess the disease burden, the patient had a PET CT (Figure 3 A-D) which showed abnormal fluorodeoxyglucose (FDG) uptake involving multiple bones (lytic and sclerotic in nature involving right rib, humerus, femur, and tibia) a soft tissue deposit involving the right side of skull with the suspicion of brain involvement, as well small nodules in both lungs. MRI of the brain (Figure 4) was done to rule out brain involvement, which showed normal brain parenchyma but an expansile lesion in the right parieto-occipital bone in keeping with metastatic disease.

To establish the diagnosis, an ultrasound-guided biopsy of the right iliac bone soft tissue mass (Figure 4) was performed which revealed metastatic renal tubulocystic carcinoma.

Treatment

After the confirmation of the diagnosis, the patient was referred for palliative radiotherapy to his painful bony lesions (right shoulder and right hip bone). He received radiotherapy dose of 20 Grays (Gy), five fractions which resulted in marked improvement in his pain. To prevent fracture in the future, the patient had prophylactic right femur dynamic hip screw (DHS) and retrograde intramedullary nailing. After completion of palliative radiotherapy, the patient was treated with sorafenib (400 mg twice a day), gemcitabine (1 g/m2 on day 1 and 8), and cisplatin (75 mg/m2) every three weekly. The patient could not tolerate his chemotherapy well, requiring dose delays and dose reductions. Due to significant dose delays, sorafenib was stopped and he continued dose-reduced chemotherapy with gemcitabine and cisplatin. He completed his six cycles of this regimen with great difficulty. Main side effects were grade III neutropenia despite filgrastim (G-CSF) support and thrombocytopenia. PET scan after second, fourth, and sixth cycles of treatment showed almost stable disease. After completion of his six cycles of chemotherapy, sorafenib was reinitiated.

## Discussion

Masson is credited with naming this tumor “Bellinian epithelioma” or “carcinoma of the collecting ducts” [2]. In 2004, Amin et al. in his case series of 29 patients coined the term as “tubulocystic carcinoma” [3]. A study by Osunkoya et al. has shown that TCRC (tubulocystic renal cell carcinoma) is distinct from CDC (collecting duct carcinoma) at the molecular level [4]. CDC is an uncommon variant of RCC that has been proven to be highly aggressive and associated with poor prognosis [3] .

Recently, TCRC was established as a rare renal cancer and considered to be less aggressive than other renal carcinomas. It occurs mostly in the fifth or sixth decade of life, with a strong male preponderance (male: female ratio, 7:1). Patients are usually asymptomatic and commonly present with abdominal pain, distension, and hematuria which are also present in almost most renal tract cancers. Most tumors have an indolent course, and rarely progress, recur, or metastasize. Only a few cases of tumor metastasizing to lymph node, liver, bone, and pleura have been reported [5] and a single case with sarcomatoid features and multiple peritoneal metastases has been reported [6]. Despite the higher grade cytological features, most patients with tubulocystic carcinoma have a favorable prognosis when surgical resection is performed at the localized stage. However, once it metastasizes, the carcinoma shows progressive and aggressive behavior.

Microscopically, it is composed of tightly packed tubules and cysts, lined by cuboidal and columnar cells containing abundant eosinophilic cytoplasm with large nuclei and prominent nucleoli. Immunohistochemistry demonstrates a poor relationship between tubulocystic renal cell carcinoma and other collecting duct tumors. Proteins of proximal convoluted tubules (CD10 and P504S), distal tubules (CK19), and intercalated collecting duct cells (parvalbumin) are expressed by these tumors. In contrast to CDC, proteins like vimentin, p53 and alpha-methyl acyl CoA racemase (AMACR) are overexpressed by these tumors [4].

The mainstay treatment for both primary and nonmetastatic disease is radical nephrectomy, but partial nephrectomy may be performed for small tumors located in the superficial renal cortex [6]. Although there is no established salvage therapy for metastatic disease, some authors have reported the effectiveness of targeted therapies. Teramoto et al. reported a case that metastasized to the lung and lymph nodes [7]. Sunitinib treatment achieved a partial response with no disease progression 12 months after nephrectomy [8].

Nevertheless, it is prudent to note that the molecular profile of both transitional cell carcinoma (TTC) and papillary renal cell carcinoma (RCC) is similar. Since the histology of CDC is similar to that of TCC, there is an analogy in the treatment approach as well. Gemcitabine with cisplatin/carboplatin has substantial activity in CDC. Several drugs including docetaxel have some activity in TCC; however, there is no established salvage therapy [9].

## Conclusions

Tubulocystic RCC is a distinct group of renal carcinoma with specific macroscopic, microscopic, and immune-histochemical findings. There is no established guideline for the management of this tumor. However, to understand its biology and ascertain true prognosis and appropriate treatment further studies are required.

## References

[1] Bhullar JS, Bindroo S, Varshney N, Mittal V (2014). Tubulocystic renal cell carcinoma: a rare renal tumor. J Kidney Cancer VHL.

[2] Anila KR, Mathews A, Augustine P, Krithika E, Jayasree K (2018). Tubulocystic renal cell carcinoma: an entity related to papillary renal cell carcinoma?. Med J Dr. D. Y. Patil Vidyapeeth.

[3] Amin MB, MacLennan GT, Gupta R (2009). Tubulocystic carcinoma of the kidney: clinicopathologic analysis of 31 cases of a distinctive rare subtype of renal cell carcinoma. Am J Surg Pathol.

[4] Osunkoya AO, Young AN, Wang W (2009). Comparison of gene expression profiles in tubulocystic carcinoma and collecting duct carcinoma of the kidney. Am J Surg Pathol.

[5] Bhullar JS, Varshney N, Bhullar AK, Mittal VK (2014). A new type of renal cancer -- tubulocystic carcinoma of the kidney: a review of the literature. Int J Surg Pathol.

[6] Indraneel B, Sher S, Vinay T (2016). Tubulocystic renal cell carcinoma: a great imitator. Rev Urol.

[7] Teramoto S, Niwakawa M, Muraoka K (2011). [Tubulocystic carcinoma of the kidney]. Nihon Hinyokika Gakkai Zasshi.

[8] Funahashi Y, Kimura Y, Kato M (2014). Metastatic tubulocystic renal cell carcinoma treated with targeted therapies. J Urol Res.

[9] Mego Z, Sycova MK, Rejlekova B (2008). Sunitinib in the treatment of tubulocystic carcinoma of the kidney. A case report. Ann Oncol.

